# Hospital and Institutional News

**Published:** 1913-10-18

**Authors:** 


### HOSPITAL AND INSTITUTIONAL NEWS.

### THE COLISEUM CHARITY PERFORMANCE.

INSTITUTIONALLY speaking, the entertainment on the evening of October 11 at the Coliseum is the event of the week. As a result, the French Hospital and Charing Cross Hospital will jointly benefit to the extent, it is hoped, of about £5,000, a truly magnificent result of Mme. Sarah Bernhardt's happy thought. To that "divine" genius, to Miss Ellen Terry, and the host of other "star" performers who freely gave their best art to the service of charity, to the management of the Coliseum, and to the numerous band of workers of many classes whose toil contributed to the success of the performance, his Majesty's message, through Sir William Carington, sums up the situation, and crowns it with the approval and congratulation which everyone else who was present feels and would express. For, indeed, the performance was a feast of good things, as was only to be expected from the dramatic profession, always ready to work its hardest for any charitable object, especially when the charity happens to be a hospital.

### THE KING'S PATRONAGE.

To their Majesties the occasion offered one more of those opportunities of showing practical sympathy with the unfortunate among their humbler subjects, which they are always ready to accept; and the Royal Family were represented in even greater strength than usual at these performances. The expression of their enjoyment of the evening contained in the King's letter was hardly needed, for it was obvious that many of the "turns" created great amusement in the Royal boxes. Mr. Harry Tate is stated to have especially delighted their Majesties; and certainly this ingenious and mirth-provoking comedian has seldom been in better form. Probably the fact that the King and Queen from the nature of things seldom get the chance of seeing artists of the variety stage enhances their satisfaction when, on occasions such as this, the very cream of the talent of that branch of the stage is presented to them in one constant succession. However that may be, the performance was an unqualified success, both artistically, socially, and financially; and the thanks of the supporters of both the beneficiary institutions to all who have been concerned, both as audience and on the stage, in this splendid work of charity are heart-felt and genuine. Elsewhere we note the inception of the scheme for opening pay wards in Charing Cross Hospital, an institution whose difficulties, if not solved, will certainly be to some extent relieved by Mme. Bernhardt's kindly and magnificent generosity.

### SOME OF THE WORKERS.

AT the conclusion of the concert the King entered the saloon which had been prepared next to the Royal box, where Miss Ellen Terry and Madame Sarah Bernhardt were presented to him by Lady Juliet Duff. The Earl of Lonsdale (chairman of the Special Appeal for Charing Cross Hospital) then presented Mr. Robin Duff and Mr. George Verity, respectively chairman and vice-chairman of that hospital, together with Mr. Edward Rochuch, representing the French Hospital in place of the Chairman, who was unfortunately prevented from attending by the sudden illness of his daughter, and Mr. A. C. Fothringham Lysons, secretary of the Special Appeal. Mr. Lysons was also warmly congratulated on the success of the entertainment by the Princess Louise, who is president of Charing Cross Hospital. It is impossible to mention all those who worked hard and unselfishly for the success of this memorable function. The two hospitals which will benefit must consider themselves under a special debt of gratitude to all those connected with the Coliseum, more especially to Mr. Oswald Stoll and Mr. Arthur Croxton, who both took a deep personal interest in the arrangements. Two ladies also should be specially mentioned—Mrs. Miriam Croxton, to whose artistic genius the decorative scheme was due, and Miss A. Grossman, who had sole charge of the bookings. The authorities are not yet in a position to give any reliable figure as to the proceeds of the entertainment, but it is estimated that a sum of £5,000 should be available for distribution between the hospitals.

### AN AMERICAN HOSPITAL WARD IN LONDON.

THE French, German, and Italian communities in London each support a hospital known by the names of their respective nationalities; but hitherto the Americans, more numerous and more affluent, probably, than any other visitors, have done nothing in a corporate way towards hospital work and support. As a matter of fact, Americans have done a lot for various of our British hospitals in which they are interested; and it would be quite erroneous to suppose that they are less generous or less charitable than the Continentals. The very nearness of their kinship with us renders them so much less strangers in our midst than those whose speech is alien, that a special hospital run by Americans is certainly not a pressing need. There is, however, no doubt that some appeal to American pride of race would be a powerful aid towards creating an interest in the work of our hospitals, and that it might induce many to subscribe who hitherto have not done so. Obvious as this sounds, now that someone has had the happy thought and translated it into deeds, we must not fail to give the credit of it to its originator, whose name has not transpired. At any rate, the good idea has resulted in the formation of a committee upon which many well-known and representative Anglo-Americans will serve; and it has been decided to endow a number of beds in the London Hospital (if possible, an entire ward), to be supported by Americans for Americans in that institution. Mr. R. P. McGrath, of the London Hospital, is treasurer, and Mr. J. P.

Coughlan, Walter House, Strand, secretary of the movement, to which we wish every success. Incidentally, we may felicitate the London Hospital also on the additional strength which the arrangement is likely to afford it.

### A PANEL PROBLEM OF TO-MORROW.

In an able public letter Mr. W. McAdam Eccles discusses in detail the difficult points involved in the distribution of the surplus funds now in the hands of the Insurance Committees. While pointing out that these funds clearly belong to the medical profession, he gives under seven heads statements of the difficulties involved equally in retaining or withholding the money. He concludes by saying that the profession is looking for a pronouncement from the State Sickness Insurance Committee of the British Medical Association, which should have foreseen the situation and outlined a policy before it occurred. He also adds, very logically, this question: "What is to be the policy of the Association should the women and children dependants of insured persons be brought into the Insurance net? This condition of things the profession may have to face in 1914. . . Perhaps the panel doctors," he concludes, "are so satisfied with their 'deal' with the Chancellor of the Exchequer that they do not wish to trouble him further; but if they are, then let them say so in no unmistakable voice, and the non-panel men will be content to stand aside and preserve as far as they can the dignity of a profession which is fast becoming a trade." It is the inactivity, or at least the silence, of the body to which Mr. McAdam Eccles alludes that has led to such a situation as that at Wandsworth.

### DIVERSION OF AN ANCIENT CHARITY.

By moving to the suburbs King's College Hospital has been forced to surrender a yearly income of £360. In Westminster there is an ancient Pest House Charity yielding £600 a year. For ages this has been allocated to King's College (which has had three-fifths) and Charing Cross (which has had two-fifths). When King's College moved from its old site in the Strand it transpired that its title to its proportion of the charity was in jeopardy. Negotiations were entered into, but now the Charity Commissioners have upheld the view of the trustees of the charity that it is essentially a local one, and as King's College, now at Denmark Hill, is no longer local to Westminster it must perforce abandon all claim to future participation. The governors of King's College, anxious not to lose such a valuable and certain source of revenue, expressed willingness to accept two-fifths so that Charing Cross could receive three-fifths instead of only two-fifths. The Charity Commissioners, however, have ruled King's College out of bounds as it were. Consequently a new scheme for dealing with the proceeds of the charity has been drawn up, and as all parties are agreed upon the suggested future allocation of the funds it may be taken for granted that henceforth Charing Cross will have three-fifths, whilst the other two-fifths will be divided amongst the following institutions: The General Dispensary, Drury Lane; the General Dispensary, Little Grosvenor Street, St. George's, Hanover Square; St. Peter's Hospital for Stone and Urinary Diseases, Henrietta Street, Covent Garden; the Royal Ophthalmic Hospital, King William Street, Strand; and the Royal Dental Hospital, Leicester Square.

### PAY WARDS AT CHARING CROSS HOSPITAL.

As was announced some considerable time ago the management of Charing Cross Hospital have decided to open free wards now closed for lack of funds for paying patients. Further details are now to hand, which show that of five closed wards three are to be opened for the reception of patients at two guineas a week—much the same charge as is made at St. Thomas's Home. It is hoped that the scheme, which will come into operation in a few months' time, will be able to pay its way; by this we presume it is meant that no cost will accrue to the hospital as a result of the experiment. If the pay-ward account is not debited with rent, taxes, share of central administration charges, and such like, it ought to pay without difficulty: if it is, we may doubt whether ends will meet. But in any event we feel sure that the authorities are acting wisely in this matter, and that in time to come other hospitals will follow the same policy. At the prices named, it is clear that there will be no competition with nursing homes or private hospitals; the benefits of the scheme are intended for, and will doubtless be appreciated by, the classes just above those for whom hospital charity is provided—namely, school teachers, clerks of both sexes, and many others in similar callings.

### THE CINEMATOGRAPH AT QUEEN SQUARE.

The magistrate at Bow Street has convicted Mr. Godfrey Hamilton, secretary to the National Hospital for the Paralysed and Epileptic, of an offence against the County Council regulations made under the Cinematograph Act of 1909. At post-graduate lectures in this hospital the cinematograph has been frequently used to illustrate the course of various diseases; and it is particularly adapted for demonstrations of nervous diseases, because the gaits and other disorders of movement associated with them can be represented so successfully in this way. The dispute between the National Hospital and the County Council arose because a subsection of the Act exempts from Council supervision private dwelling-houses to which the public are not admitted. The Council claimed that the Act and its regulations applied to these lecture-demonstrations; the National Hospital claimed that it did not, and refused to conform to all the County Council's regulations. The magistrate found against Mr. Hamilton, and ordered fines amounting, with costs, to £8 3s. As this money will presumably have to come eventually out of the purse of the National Hospital, and as there was a distinct doubt as to the exact legal interpretation of the Act, we suggest that a nominal fine without costs would have fitted the case better.

### THE BRITISH LYING-IN HOSPITAL.

Some time ago the decision to remove the well-known maternity charity in Endell Street was made known officially, and now the actual sale of the old premises is approaching. The *Westminster Gazette* aptly reminds us of some of the associations of the neighbourhood, which is a piece of old London being rapidly transformed out of recognition. In 1749 the hospital was established in Brownlow Street, then part of the estate of the family of that name, who had their town house and garden there. Exactly a hundred years later the hospital was transferred to Endell Street, not far away, wherein was a celebrated " bath " supplied from a medicinal spring. This spa enjoyed great favour during the early part of the eighteenth century, and it is stated that Queen Anne was one of its patrons. As lately as 1851 the spring received honourable mention in a " View of London." It is described as powerfully tonic with ferruginous constituents, and the inhabitants of the neighbourhood extolled its virtues for rheumatism and kindred disorders. By the late seventies the spring had ceased to flow, and the upper part of the old bathhouse, which had a " lofty French groined domed roof," housed a blacksmith's shop.

### PRODUCTIVE SOURCES OF INCOME.

We note with pleasure a series of successful fancy-dress parades and galas in Leicestershire for the funds of the County Hospital. The management of this institution is fully alive to the value of arousing the activities and enthusiasm of the Leicestershire populous centres and their zeal for the welfare of the sick. The Leicester Royal Infirmary takes great care to give publicity in each year's report to the cost of treatment of patients from every town and village. This return at times shows interesting comparisons between cost of treatment and income received; but if this year's efforts are a criterion there will soon be an end to the anomaly of districts sending less than the cost of treatment of patients. Many efforts have been organised for the institution recently. Coalville from its first parade and gala sent a contribution of £150, after carrying £45 forward to next year's efforts; Bagworth, from a similar effort, sent £80; Moira and Donisthorpe £76, an initial effort at Ibstock £75, and Mountsorrel collected £29 2s., Broughton Astley £31 10s., Sileby Adult Schools £27 14s., Countesthorpe £26 5s., Barlestone £24 16s. 9d. In addition, Pegg's Green hopes to send its usual contribution of about £30 from a flower show, and Thringstone sent from a similar effort £14. The help thus given is greatly to the credit of the parishes concerned, and when it is remembered that some of them are small colliery parishes, the residents of which contribute systematically to the work of the hospital through their colliery funds, the result is all the more gratifying. We hope the number of such efforts will gradually extend throughout Leicestershire and result in a permanent addition to the income of Leicester's large hospital.

### CHANGES AT THE GENERAL HOSPITAL, WOLVERHAMPTON.

To the general regret of his friends and the friends of the Wolverhampton General Hospital Mr. J. S. Neil has been obliged for reasons of health to resign the post of house governor and secretary to that institution and to go abroad. He has held this appointment amid general approval for seven years, a term during which very important and arduous work in the development of the institution has been necessary. Mr. Neil is well known in the institutional world as the founder of the Hospital Officers' Association, of which he was honorary secretary and treasurer in 1902-3. In his place the board have appointed Mr. W. H. Harper, who is a Wolverhampton man long connected with the General Hospital. For twelve years he has been on the staff, first as collector and then as assistant secretary. Mr. Harper will, it is stated, retain the secretaryship of the local Hospital Saturday Fund Committee; it is largely through the energy which he has infused into this fund that it is now on such a satisfactory footing, and Mr. Harper has especially distinguished himself in interesting the workpeople of the locality in the collections. He is personally popular with them as well as with the patrons of the hospital, and the ungrudging service he has long rendered to the hospital makes his selection for the secretaryship most opportune.

### BENEFACTIONS TO THE SOUTH WARWICKSHIRE HOSPITAL.

A splendid response has been obtained to the challenging example set by Mrs. B. W. Jones, of Brookhurst, Leamington, in giving £100 to the Warneford and South Warwickshire Hospital, and in promising a further £100 if £400 were raised before September 30. Actually, not £400, but £800 was obtained, £300 of which comes from the family which set the matter on foot. The list shows that Mrs. Jones was as good as her word in giving £200, and Mr. Jones gave £100. Three more ladies gave £100 each. There was one donation of £50, and two of £25, as well as two collections which realised about £42; and smaller sums made up the balance. It will be seen that the total sum was contributed almost wholly by a small number of wealthy people, chiefly inhabitants of Leamington. It is satisfactory to know that such an excellent charity possesses such generous friends, but it also behoves the people of the district to subscribe as well in proportion to their means, and not to leave the whole burden on a few shoulders. We congratulate the hospital on the very welcome addition to its funds.

### THE SECRETARYSHIP OF THE DAVID LEWIS HOSPITAL, LIVERPOOL.

The successor of Mr. Joseph Unsworth as secretary superintendent of the David Lewis Northern Hospital, Liverpool, is Mr. H. Levey. As we announced in our columns on July 12 last, Mr. Unsworth retired after forty-five years' service, a period which can have few rivals, even in the long records of work which are fairly common in the institutional world. Mr. H. Levey, the new secretary superintendent, has already done work for the David Lewis Hospital, having acted as its collector for some time past. Liverpool has always been an active centre of hospital work, and though Mr. Levey enters on his secretarial duties at an anxious time, when the cry of falling legacies is often heard, such emergencies always prove an inspiration to the best man, and he may look forward to a responsible and, therefore, interesting if onerous period of superintendence.

### THE EAST SUFFOLK AND IPSWICH HOSPITAL.

We congratulate the management upon the appearance and get-up of the annual report of this institution for the year ended May 31 last. The illustrations are effective, in the sense that they are so well reproduced as to be readily understood, so that any expert person can grasp each of the many points they exhibit and the lessons they teach. The accounts are printed and presented in the best possible form, and there is proof of the sound system of business which evidently permeates the management. Some of the statistical tables have a special interest from their fulness and clearness. They closely follow the recognised standard and give much information in the briefest possible space. They are so presented as to enable a comparison to be readily made between the relative cost of the material items of one year with another. The table relating to the washing done on the hospital premises is good, and we should like to see it generally adopted by other hospitals and included in their reports. We are glad to see that the Secretary has been wise enough to include in his statistical tables the comparative statement of income and expenditure for a term of years. This statement fills but one page in the report, but its importance becomes apparent when examined, and its publication shows confidence in the soundness of the management and the economical administration of this hospital. We shall hope to have the pleasure of inspecting it in the near future.

### NEMESIS OF THE SMALL ISOLATION HOSPITAL.

Of isolation hospitals provided by local authorities out of current rates some are frame buildings of iron and wood such as are kept in stock by the makers of portable buildings, or locally-made structures of a similar sort; some are existing dwelling-houses or other buildings purchased or hired; others again are tents or huts purchased for the occasion and intended to be erected on any site that may be available. There are thus two classes of small hospitals in existence. There are the small permanent hospitals which have been designed for use as such and furnished with the necessary equipment; and there are the temporary or extemporised hospitals, provided at a minimum of cost, and often not until an emergency calling for their use has arisen. This is really the practical argument against extemporised or temporary structures for the isolation of infectious diseases.

### HOSPITALS IN WEST AFRICA.

We are apt to forget that hospitals are needed on the frontiers of civilisation as elsewhere; yet, according to the last Colonial Office report on Gambia, in West Africa, there are two hospitals, one at Bathurst and one at MacCarthy Island. The former has accommodation for four first-class, eleven second-class, and ten third-class male patients, and fifteen female patients, including two cots for children. There are also four rooms for isolation cases. The wards for males and females are in separate buildings. The hospital, opened in 1911, at MacCarthy Island is in charge of a European medical officer. It contains a ward for Europeans and three native wards. Sixty-six in-patients and 1,356 out-patients were treated during the year. There is also a contagious diseases hospital, situated about two miles from Bathurst. Four mild cases of smallpox, all of which recovered, were treated in this building during the past year. The medical establishment consists of a senior medical officer and four other medical officers, all of whom are members of the West African Medical Staff. The senior and two other medical officers are resident in Bathurst, one is stationed at MacCarthy Island, and one is a travelling medical officer in the Protectorate. The total number of in-patients treated in the Bathurst Hospital during the year was 529. Thirty-three patients died in hospital. The number of cases treated in the out-patients' department was 7,895. Of the thirty-three deaths, four were from broncho-pneumonia, three from bronchitis, while chronic malaria, debility, pneumonia, congestion of the lungs, abscess, general injuries, and valvular mitral were each responsible for two deaths. Institutional workers who have the gift of reading between the lines will gain from these facts some idea of the work of their colleagues in Gambia.

### MR. ST. LOE STRACHEY'S WEATHER-BOARD CONSTRUCTIONS.

The new design of Mr. J. St. Loe Strachey's recently built cottage at Merrow Common, near Guildford, the offspring of a challenge if we remember aright, has received the approval of the local medical officer of health. The cottage, which is made of weather-board, is thus reported on by Dr. R. W. C. Pierce, medical officer of health to the Guildford Rural District Council. The floor boards on the ground floor, which are laid in pitch upon cement concrete, he states, are satisfactorily protected against damp; while the wooden walls, he adds, are likely to remain much more dry than those more familiar to us to-day, which are made of cheap porous brick. While "passing" the cottage as satisfactory from the health point of view, Dr. Pierce does not recommend its general adoption in rural districts. Granting the temporary nature of Mr. Strachey's cottage, the question has to be considered whether or not it would pay better, both for the occupier as well as the builder, to replace at regular intervals or to construct a substantial building. Until this economic factor is decided our knowledge of the value of Mr. Strachey's interesting experiment remains incomplete; though institutional managers who often require temporary shelters and similar structures will be glad to hear the details of a form of construction that might be adaptable to purposes of theirs.

### SALE OF A MEDICAL PUBLISHING BUSINESS.

Medical publishing has become during the last ten years a much more strenuous affair than it was wont to be. Competition is now so keen that only the most up-to-date methods will suffice to ensure continued success. In evidence of which the curious will find an advertisement in the *Lancet* offering for sale by tender, as a going concern, the "old-established London medical publishing business of Rebman, Limited." This firm gained a gold medal at the recent International Congress of Medicine; but apparently misfortune has overtaken it. The property offered for sale includes not only the goodwill of the business and the premises in Shaftesbury Avenue, where it is carried on, but also many copyrights, and a "valuable contract for the supply of calf lymph," a curious appendage of a publishing business. If memory serves us, this firm has been prominently identified with the sale of Continental and American medical works. Possibly the announcement referred to is due to the increasing preference of British students and doctors for text-books by British authors. We note that the advertisement asks for bids, but discloses no details of the profits or of any other matter which would be of importance to intending purchasers.

### TWO LARGE LEGACIES FOR HOSPITALS.

The Leeds General Infirmary and the Bath Royal Mineral Water Hospital will ultimately benefit very largely under the will of the late Mr. John Albert Nunneley, M.B., M.R.C.S., who was consulting surgeon to the former institution and a governor of the latter. For thirty years Mr. Nunneley was ophthalmic surgeon to the Leeds General Infirmary, and he had also been senior surgeon to the Leeds Eye and Ear Infirmary, to which no bequest is allotted. Mr. Nunneley devised his property to his wife, and on her death, subject to some family legacies and to a sum of £100 left to the United Kingdom Beneficent Association, the residue is bequeathed as to two-thirds to the Leeds General Infirmary for investment, and as to one-third to the Royal Mineral Water Hospital for the same purpose. The gross value of the property is £55,556, with net personalty £49,605. Before deduction of legacy duty the amount available for the two hospitals will be nearly £45,000.

### A FORTUNE FOR HOSPITALS.

Another series of munificent legacies to hospitals was announced on October 13, when particulars were made public of the testamentary dispositions of Miss Anna Jane Arden, a cousin of Lord Burton. This lady has left the whole of her fortune, amounting to some £67,000, to be equally divided among the following institutions: St. George's Hospital, St. Thomas's Hospital, St. Mary's Hospital, Great Ormond Street Hospital for Children, the Putney Hospital for Incurables, the Queen Square Hospital for the Paralysed and Epileptic, the Brompton Consumption Hospital, the Reedham Orphanage at Purley, the Margate Deaf and Dumb Asylum, the Orphan Asylum at Bagshot, the Universal Beneficent Society, the London Surgical Aid Society, the County of Stafford Police Court Mission, and the Mission to Deep-Sea Fishermen. After payment of estate and legacy duties, the institutions mentioned will receive each about £4,000.

### THE MOTHERS' HOSPITAL.

This is the name given to the Salvation Army's Maternity Home in connection with the Women's Social Work Section of the Army. It is interesting to note that rather more than a year ago the first of a series of what may be known as mothers' hospitals was opened at Islay, Alberta, Canada. The shacks on the prairie do not afford adequate accommodation or provide the necessary comforts and conveniences, with the medical service, by which maternity should be safeguarded. The distances are great, and a hospital like that at Islay, to which the mothers can be admitted, represents a great and much-needed reform in Canadian organisation. It is an interesting fact that whereas the Salvation Army is to open the first mothers' hospital in Lower Clapton Road to-day an energetic and capable lady, Miss Ada B. Teetgen, should have mainly contributed to the formation and opening of the first mothers' hospital in Canada more than a year ago. We hope that every district throughout agricultural Canada may speedily have a similar hospital to that at Islay, for their establishment would remove many hardships and dangers which beset the young mother there under existing conditions. The Mothers' Hospital in Clapton has been well thought out, infinite pains have been taken to secure a thoroughly practical and up-to-date plan, and the institution, of which we propose to give an account and plans next week, offers points of interest which should ensure a large gathering of interested persons at the opening on Saturday the 18th instant.

### DEATH OF MR. G. B. STOCKER.

The announcement of the death of this well-known medical agent at the age of fifty-seven years will come as a shock to the many hundreds of medical men who have at one time or other had business dealings with him, and to those institutional secretaries who have availed themselves of his service to secure house officers and *locum tenentes*. Technically his agency was known as the Scholastic, Clerical, and Medical Association, of which he had been managing director for thirty years; but to his large circle of medical clients it was always known as "Stocker's," so much was he its driving force. The great success which he achieved was due in the main to the combination of business acumen and absolute straightforwardness which he brought to bear on all his clients'

affairs. There are, of course, other medical agents who enjoy and deserve the confidence of their clients, but none of them had quite the prestige which attached to Mr. Stocker. The gap created by his death will doubtless be filled in time, but there must be a great many men now in practice to whom the removal of such an experienced and trusted friend will at first seem irremediable.

### WALTHAMSTOW HOSPITAL'S LOSS.

WALTHAMSTOW HOSPITAL loses this month its oldest honorary medical officer in the person of Dr. C. H. Wise, who from the time of this institution's inception as a general hospital has taken a keen, whole-hearted, and active interest in its work. There is a particular sadness in the loss in that the resignation comes as the result of ill-health. Dr. Wise is an M.D., L.R.C.P. (Lond.), M.R.C.S. (Eng.); he is a barrister-at-law, J.P. for the County of Essex, and was for some time deputy coroner for the county district. He has also held the position of honorary medical officer to the Walthamstow Dispensary.

### HARVEY, DARWIN, HUXLEY.

THE Gresham Lectures for the present term will be four in number, given as usual by Dr. F. M. Sandwith, the Gresham Professor. The first will be delivered on October 28 and will deal with the career of William Harvey, the discoverer of the circulation of the blood and one of those men who were too far in advance of their times to receive in life the honour which posterity awards them. The second lecture is on October 29, and its subject is to be the life story of Charles Darwin; and the third, on the following day, takes Thomas Huxley for its subject. All three of these illustrious scientists were medical men by training, but each of them abandoned practice, though Harvey did not, like Darwin and Huxley, do so early in his career. The last lecture, on October 31, will be devoted to lantern slides bearing on the subjects of the three previous lectures, and some relics of the three great scientists will be exhibited. The lectures are free to the public, and are delivered at 6 P.M. at the City of London School, Victoria Embankment, E.C., close to Blackfriars Bridge.

### BROKEN WINDOWS IN HARLEY STREET.

SOME few years ago an enterprising reporter walked down Harley Street counting the number of open windows compared with those shut. The result was not very complimentary to the inhabitants, whose precepts seemed to be more hygienic than their practice. We fear that it was not a desire to admit more fresh air into the houses of Harley Street and Wimpole Street which led some "wild women" to stone the residences of seven or eight doctors on October 10, including those of the Presidents of two Royal Colleges. Their own statements represent these outrages upon decency as retaliation for the forcible feeding of two prisoners in Holloway Gaol, a specimen of the irrational and confused mental processes of these pests of society which is much on a par with the rest of their propaganda. The moral of these performances we do not propose to draw here; but the bulk of our population, female as well as male, has been drawing it for the last two or three years. Meanwhile we can only offer our sympathies to the victims of these maniacal outbursts.

### A CRIMEAN AND INSTITUTIONAL VETERAN.

THE little band of medical Crimean veterans has suffered still further diminution by the death of Mr. Henry John Thorp, who has died at Ipswich of pneumonia, at the age of eighty-one. Educated at Guy's Hospital, he qualified as M.R.C.S. in 1854, and was surgeon on the *Perseverance* during the Crimean campaign. Afterwards he practised in London, and was surgeon to the Royal South London Dispensary.

### THE HISTORY OF MEDICINE.

THE Royal Society of Medicine is taking a step which can command nothing but approval in arranging for lectures at No. 1 Wimpole Street on medical history, to be given by experts and open to the public on presentation of a visiting card. The first was announced for October 10, by Professor Morris Jastrow (Pennsylvania University) on "Babylonian Medicine"; the second is by Professor Elliott Smith on October 30, on "The Contributions of Ancient Egypt to the History of Medicine"; and the third by Professor Richard Caton on December 17, on "Health Temples of Ancient Greece and the Work carried on in them." All the lectures are at 5 P.M., and entirely free, subject to the one condition noted above. In thus popularising medical history the Royal Society of Medicine is doing good work; and in view of the widespread interest taken by the general public in the proceedings of the historical section of the recent International Congress of Medicine the decision to admit laymen is excellent.

### THIS WEEK'S DRUG MARKET.

PRICE changes have been few during the week and business has been quiet. Cod-liver oil is a very dull market and there is a slight downward tendency in price; it is not improbable, however, that when the winter demand sets in this tendency will be reversed. The price of quinine is still unchanged and, judging from the quiet demand, buyers do not seem to be expecting any further advance in makers' quotations just at present. Carbolic acid has a slightly lower tendency. In consequence of a further advance in the price of citric acid, quotations for potassium citrate, sodium citrate, and iron and quinine citrate have been raised. Oil of cloves is slightly cheaper. The downward movement in menthol prices has been checked, but it is not improbable that there will be a further decline before long. Ergot of rye is somewhat lower in price. Opium and its alkaloids are without change. Orange peel is dearer owing to scarcity.

